# Multicolor fluorescent *in situ *hybridization to define abutting and overlapping gene expression in the embryonic zebrafish brain

**DOI:** 10.1186/1749-8104-6-10

**Published:** 2011-04-05

**Authors:** Gilbert Lauter, Iris Söll, Giselbert Hauptmann

**Affiliations:** 1 Department of Biosciences and Nutrition, Karolinska Institutet, SE-141 83 Huddinge, Sweden

## Abstract

**Background:**

In recent years, mapping of overlapping and abutting regulatory gene expression domains by chromogenic two-color *in situ *hybridization has helped define molecular subdivisions of the developing vertebrate brain and shed light on its basic organization. Despite the benefits of this technique, visualization of overlapping transcript distributions by differently colored precipitates remains difficult because of masking of lighter signals by darker color precipitates and lack of three-dimensional visualization properties. Fluorescent detection of transcript distributions may be able to solve these issues. However, despite the use of signal amplification systems for increasing sensitivity, fluorescent detection in whole-mounts suffers from rapid quenching of peroxidase (POD) activity compared to alkaline phosphatase chromogenic reactions. Thus, less strongly expressed genes cannot be efficiently detected.

**Results:**

We developed an optimized procedure for fluorescent detection of transcript distribution in whole-mount zebrafish embryos using tyramide signal amplification (TSA). Conditions for hybridization and POD-TSA reaction were optimized by the application of the viscosity-increasing polymer dextran sulfate and the use of the substituted phenol compounds 4-iodophenol and vanillin as enhancers of POD activity. In combination with highly effective bench-made tyramide substrates, these improvements resulted in dramatically increased signal-to-noise ratios. The strongly enhanced signal intensities permitted fluorescent visualization of less abundant transcripts of tissue-specific regulatory genes. When performing multicolor fluorescent *in situ *hybridization (FISH) experiments, the highly sensitive POD reaction conditions required effective POD inactivation after each detection cycle by glycine-hydrochloric acid treatment. This optimized FISH procedure permitted the simultaneous fluorescent visualization of up to three unique transcripts in different colors in whole-mount zebrafish embryos.

**Conclusions:**

Development of a multicolor FISH procedure allowed the comparison of transcript gene expression domains in the embryonic zebrafish brain to a cellular level. Likewise, this method should be applicable for mRNA colocalization studies in any other tissues or organs. The key optimization steps of this method for use in zebrafish can easily be implemented in whole-mount FISH protocols of other organisms. Moreover, our improved reaction conditions may be beneficial in any application that relies on a TSA/POD-mediated detection system, such as immunocytochemical or immunohistochemical methods.

## Background

The complex functional and anatomical organization of the vertebrate forebrain and its dynamic development led to a variety of interpretations of its basic organization. However, in the past decades, the examination of forebrain-specific regulatory gene expression patterns supported the development of a prosomeric concept of forebrain organization [1-3]. The characterization of prosomeres was largely supported by the identification of gene expression domains that predict and are consistent with proposed prosomeric territories and borders [4]. Thus, the molecular characterization of prosomeres strongly relied on identification of abutting or overlapping gene expression domains. In zebrafish, chromogenic two-color whole-mount *in situ *hybridization allowed the direct visualization of expression domains of two genes in different colors in the same embryo [5-9]. The establishment of this method greatly facilitated the correlation of forebrain gene expression domains with each other and, in agreement with the prosomeric model, led to the identification of transverse and longitudinal subdivisions in the zebrafish forebrain [10-12]. Two-color whole-mount *in situ *hybridization has also been used to localize distinct neuronal cell groups, such as catecholaminergic and corticotropin-releasing hormone neurons, to prosomeric subdivisions [13,14].

In the original zebrafish protocol, digoxigenin- and fluorescein-labeled nucleic acid probes were simultaneously hybridized and subsequently visualized in two consecutive rounds of antibody-alkaline phosphatase conjugate-based detection using Fast Red and BCIP/NBT as the chromogenic substrates, respectively [6,9]. However, overlapping or colocalized expression is often difficult to resolve by chromogenic two-color *in situ *hybridization because of lower second round detection sensitivity, masking of the lighter red signal by the darker blue color precipitate, and lack of three-dimensional visualization possibilities.

These limitations may be overcome by fluorescent *in situ *hybridization (FISH), which offers selective detection of different transcripts at high spatial resolution. In combination with confocal imaging, the advantages of digital image processing and visualization can be fully exploited (for example, colocalization analysis, optical sectioning, three-dimensional reconstruction) [15]. Current whole-mount FISH protocols apply horseradish peroxidase (POD) and fluorescent tyramide substrates for signal amplification [16-19]. Despite the increased sensitivity through tyramide signal amplification, POD substrate turnover is still limited by the relatively short reaction time compared to alkaline phosphatase, so that less abundant mRNA species may be still difficult to detect.

In zebrafish embryos introduction of the tyramide signal amplification (TSA) system into multiplex FISH applications has been difficult, not least due to the large, hydrophobic yolk where the substrate can be easily trapped [20,21]. In order to precisely define overlapping and abutting gene expression domains of a large variety of genes in the embryonic forebrain, we were in need of developing an optimized FISH protocol that allowed the visualization of low copy number transcripts. By addition of the viscosity-increasing polymer dextran sulfate to the hybridization and TSA-POD reaction and application of substituted phenol compounds as POD accelerators, we optimized hybridization and POD-TSA reaction conditions to obtain dramatically improved signal sensitivities and signal-to-noise ratios. The use of highly effective bench-made tyramide substrates further increased signal intensities. These measurements were prerequisites for multicolor fluorescent detection of up to three different transcripts at cellular resolution. Differently hapten-labeled antisense RNA probes were simultaneously hybridized and sequentially detected in up to three rounds of anti-hapten antibody and POD-TSA detection. For multicolor detection, it was essential that the POD was effectively inactivated after each detection cycle by glycine-HCl treatment.

## Results and discussion

### Effects of viscosity-increasing polymers and substituted phenol compounds

**Figure 1:**
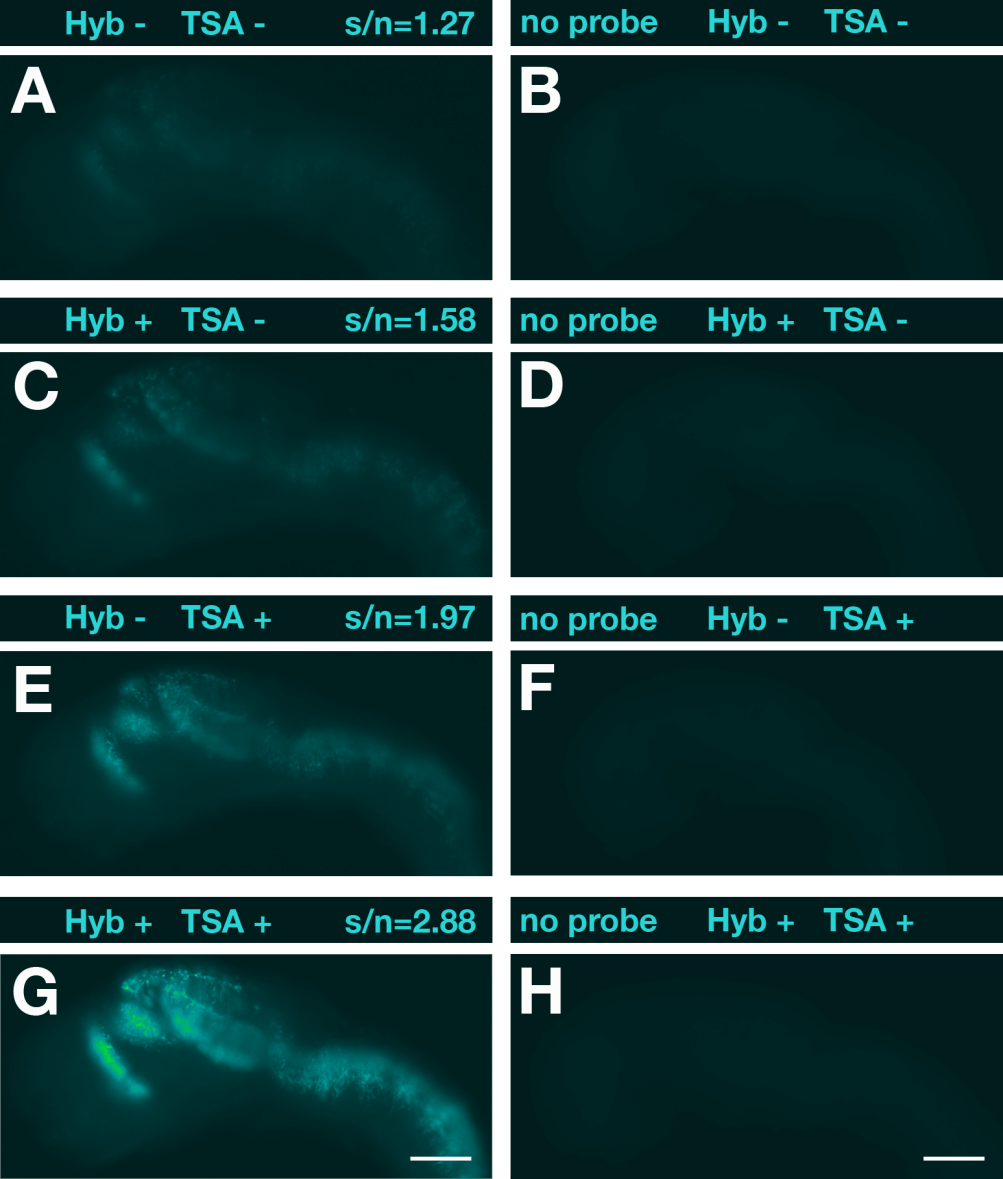
**Optimization of hybridization and POD reaction by dextran sulfate**. **(A, C, E, G) **Lateral views of 1-dpf zebrafish embryos hybridized with a digoxigenin-labeled antisense RNA probe specific for *dbx1a *are shown with anterior to the left. Above each panel signal-to-noise ratios (s/n) are indicated and whether dextran sulfate was added (+) or not (-) to the hybridization buffer (Hyb) and/or TSA reaction. **(B, D, F, H) **Embryos hybridized without probe were used as negative controls to assess background noise. Black-and-white pictures were recorded with identical exposure times using an Orca digital camera (Hamamatsu) on an Axioplan II microscope (Carl Zeiss). Images were false-colored with help of the ImageJ software and no further adjustments or other image processing was performed. Scale bar = 100 μm.

Despite using the TSA system for signal enhancement in FISH applications, the results can be dissatisfying with respect to signal sensitivity and signal-to-noise ratio. For optimal *in situ *detection of transcripts, we therefore attempted to improve the efficiency of crucial steps of the FISH procedure. To increase local probe concentration during hybridization, we added a viscosity-increasing polymer to the hybridization buffer. We hybridized 1-dpf (days post-fertilization) zebrafish embryos with a *dbx1a*-specific RNA probe under identical conditions except for the lack or inclusion of dextran sulfate. We found that addition of 5% dextran sulfate (molecular weight >500,000) during hybridization led to a significant increase in signal intensity (Figure 1A, C). Similar results were obtained with several other RNA probes tested (data not shown).

Next we examined the effects of dextran sulfate when included in the TSA-POD reaction. Addition of dextran sulfate significantly enhanced the specific signal intensity compared to the fluorescent signal obtained without polymers (Figure 1A, E). The effect was dose dependent with increasing dextran sulfate concentration (5%, 10%, 20%), resulting in increased signal intensity (data not shown). Since high amounts of dextran sulfate resulted in negative osmotic effects eventually leading to deformations in morphology, we decided to routinely use a 2% concentration for the POD reaction. The strongest signal enhancement was obtained when including dextran sulfate in both the hybridization and TSA-POD reaction (Figure 1G). As a negative control, we processed embryos for FISH without adding a probe to the hybridization mix (Figure 1B, D, F, H). Addition of dextran sulfate did not increase background noise (autofluorescence). Thus, the substantial increase in signal intensity resulted in a significantly improved signal-to-noise ratio (Figure 1G). The signal enhancement through addition of dextran sulfate is most likely the result of macromolecular crowding effects on the conditions of the hybridization and TSA reactions [22].

Previous studies indicated that addition of polyvinyl alcohol, another viscosity-increasing polymer, to the POD reaction medium could improve localization sharpness and sensitivity [23]. In our experiments, however, addition of 10% polyvinyl alcohol (molecular weight 85,000 to 124,000) to the TSA-POD reaction did not result in a stronger specific fluorescence signal (data not shown). Similarly, imidazole has been described to increase the sensitivity of POD-catalyzed reactions [24]. The use of 10 mM imidazole as described in [24] did not increase the fluorescent signal obtained by the POD-TSA reaction (data not shown).

**Figure 2:**
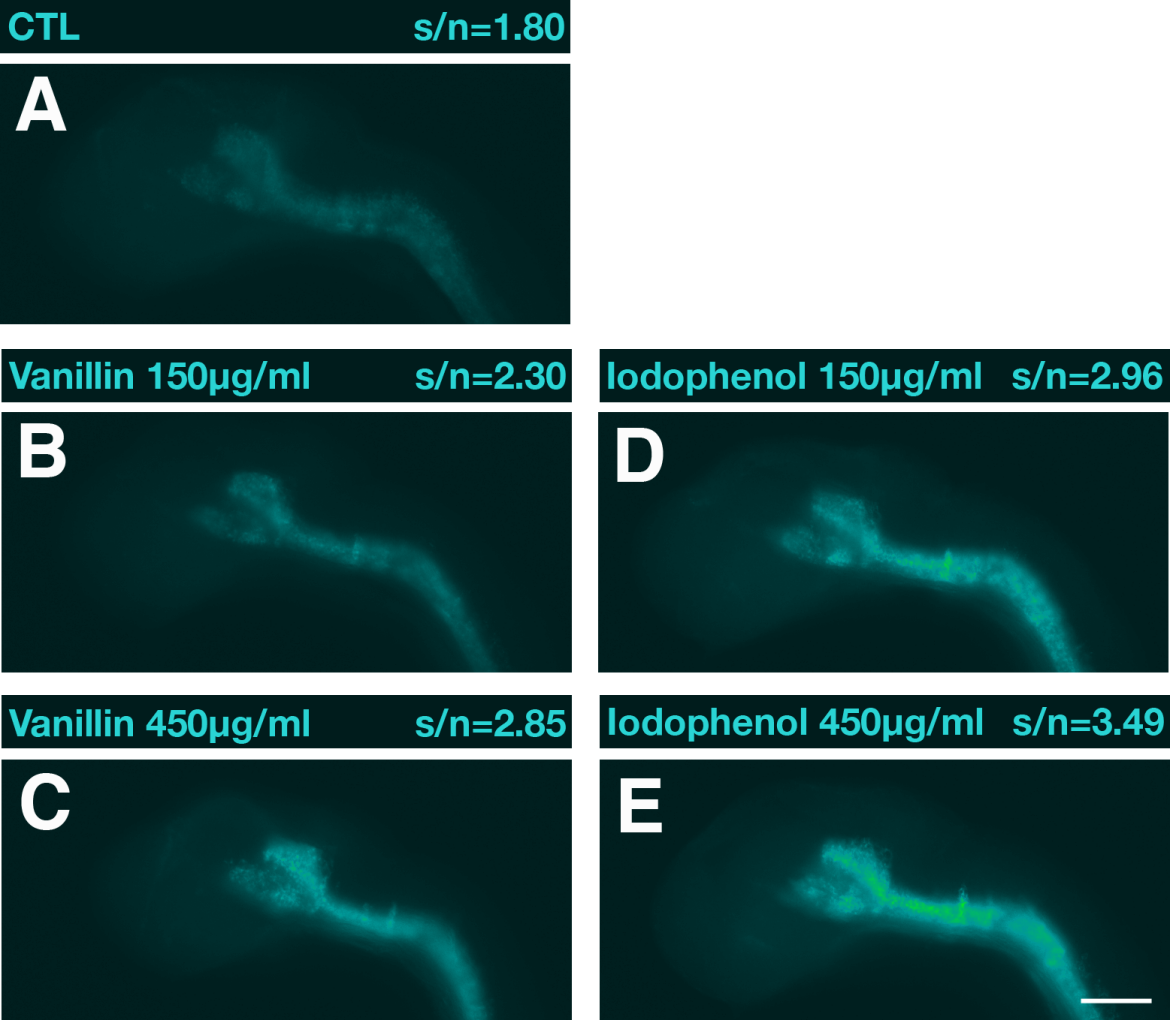
**Positive effects of substituted phenol compounds on the TSA-POD reaction**. Lateral views of 1-dpf zebrafish embryos hybridized with a digoxigenin-labeled antisense RNA probe specific for *nkx6.1 *are shown with anterior to the left. **(A) **Control (CTL); **(B, C) **vanillin added; **(D, E) **4-iodophenol added. In all embryos shown, dextran sulfate was included in the hybridization and TSA-POD reaction. Above each panel signal-to-noise ratios (s/n) and compound concentrations are indicated. Black and white pictures were recorded with identical exposure times. Images were false-colored with help of the ImageJ software and no further adjustments or other image processing were performed. Scale bar = 100 μm.

Next we tested whether we could obtain a significant rate enhancement of the fluorogenic reaction by addition of an appropriate accelerator compound [25]. We examined whether the substituted phenol compounds 4-iodophenol and vanillin could be used as accelerators of the TSA-POD reaction. Zebrafish embryos at 1 dpf were hybridized to a RNA probe specific for *nkx6.1 *and further processed in parallel in an identical fashion except for the addition of 4-iodophenol or vanillin to the TSA-POD reaction. The addition of vanillin (Figure 2B, C) and 4-iodophenol (Figure 2D, E) in the range 150 to 450 μg/ml resulted in a dose-dependent, significant increase in specific signal intensity compared to accelerator-free reactions (Figure 2A). 4-Iodophenol turned out to be the most potent accelerator. Corresponding strong rate enhancements were obtained when trying other RNA probes for testing of the effects of these compounds (data not shown).

### Application of bench-made TSA substrates

**Figure 3:**
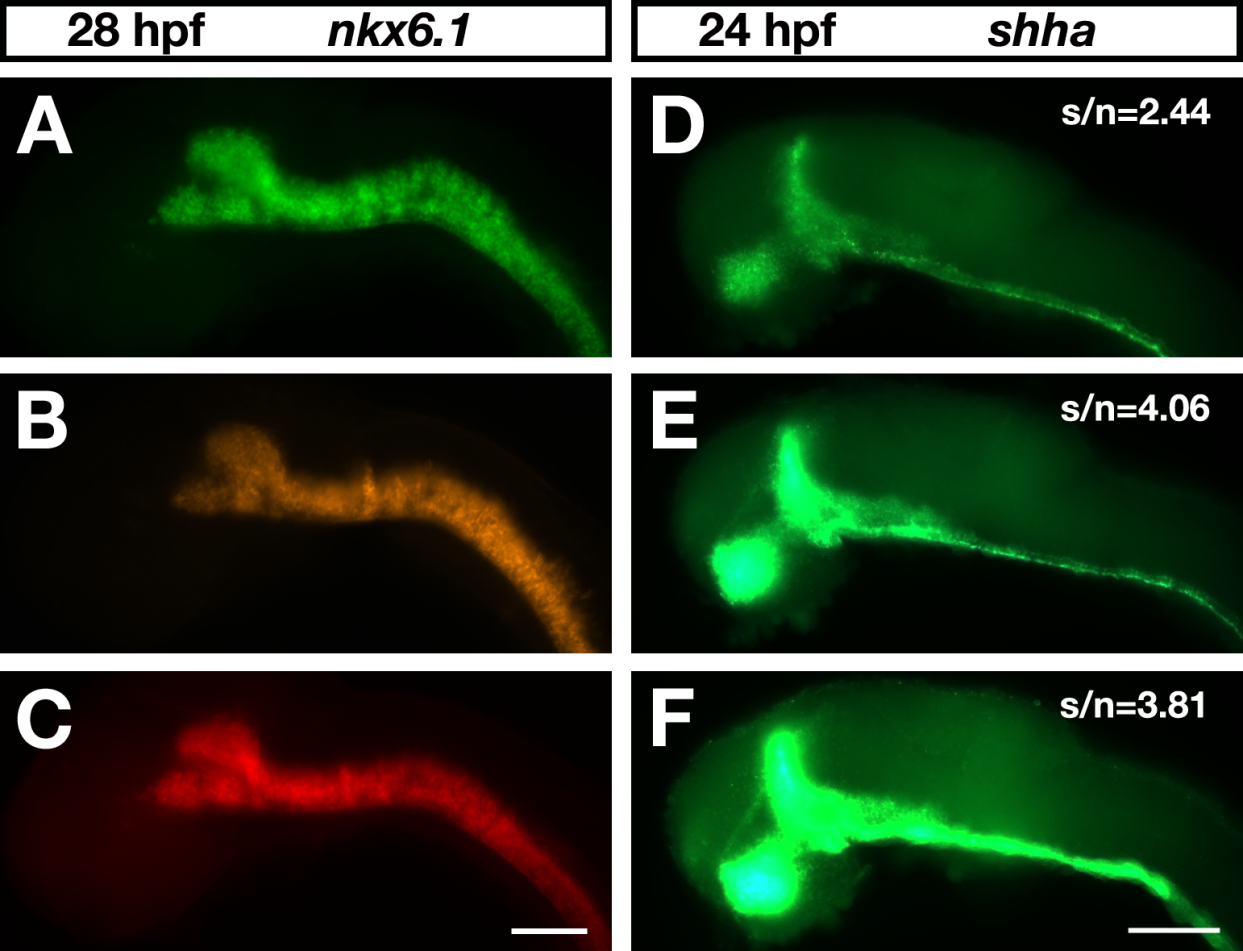
**Bench-made fluorescent tyramides applied in zebrafish whole-mount FISH**. Embryos were viewed on an Axioplan II microscope (Carl Zeiss). For excitation, a mercury burner (HBO 103 OSRAM) was used. Lateral views of zebrafish brains are shown with anterior to the left. Scale bar = 100 μm. (**A-C**) Zebrafish embryos at 28 hpf hybridized to a digoxigenin-labeled *nkx6.1 *antisense RNA probe. Images were captured with an Orca digital camera (Hamamatsu). As POD substrates, three different bench-made fluorogenic tyramides at a 1:250 dilution were used: (A) FAM-tyramide (exposure time, 100 ms; Chroma-filter 41001); (B) TAMRA-tyramide (exposure time, 4 ms; Chroma-filter 41002b); and (C) DyLight633-tyramide (exposure time, 120 ms; Chroma-filter 41008). (**D-F**) Zebrafish embryos at 24 hpf hybridized to a digoxigenin-labeled *shha *antisense RNA probe. Images were recorded with an Axiocam digital color camera (Carl Zeiss) using identical exposure times. Transcript distribution was visualized with (D) fluorescein-tyramide from Perkin Elmer (SAT701B001EA) at a 1:100 dilution, (E) bench-made FAM-tyramide at a 1:250 dilution, or (F) a 1:100 dilution.

For *Xenopus *embryos, it was reported that commercial fluorescent tyramide substrates did not work in whole-mount FISH [19]. Similarly, we found that the use of commercial TSA substrates under standard conditions without viscosity-increasing polymers and rate accelerators resulted in poor fluorescence signal intensities in zebrafish whole-mount FISH. We therefore generated our own set of tyramides. Tyramide substrates can be rapidly synthesized by coupling amine reactive succinimidyl esters to tyramine through stable amide bonds [26]. We used this simple one-step chemical reaction to generate 5-(and-6)-carboxyfluorescein (FAM), 5-(and-6)-carboxytetramethylrhodamine (TAMRA), and DyLight633 labeled tyramides and tested their performance in 28-hpf (hours post-fertilization) zebrafish embryos hybridized to a *nkx6.1 *antisense RNA probe (Figure 3A-C). The expression pattern of *nkx6.1 *in the fore-, mid- and hindbrain could be clearly visualized with each of the synthesized fluorescent tyramide conjugates (Figure 3A-C). The exposure times to achieve similar signal intensities were 100 ms, 4 ms and 120 ms for FAM-, TAMRA- and DyLight633-tyramide, respectively, indicating that TAMRA was most sensitive.

To compare the performance of bench-made and commercial tyramides, 24-hpf zebrafish embryos were hybridized with a digoxigenin-labeled antisense *shha *RNA probe (Figure 3D-F). Expression of *shha *in ventral fore- and midbrain, zona limitans intrathalamica and floorplate could be visualized with both bench-made FAM-tyramide (Figure 3E, F) and commercial fluorescein-tyramide (Figure 3D). However, at the manufacturer's recommended dilution of 1:100 the commercial tyramide conjugate provided relatively weak or moderate signal intensity (Figure 3D) while under identical conditions the bench-made tyramide conjugate led to a very strong signal (Figure 3F). Even when used at higher dilutions the bench-made tyramides generated a significantly stronger signal intensity leading to a dramatically improved signal-to-noise ratio (Figure 3E). Since there is no information about the tyramide concentration provided by the supplier, we assume that commercial tyramide substrates may be highly diluted and therefore less effective.

**Figure 4:**
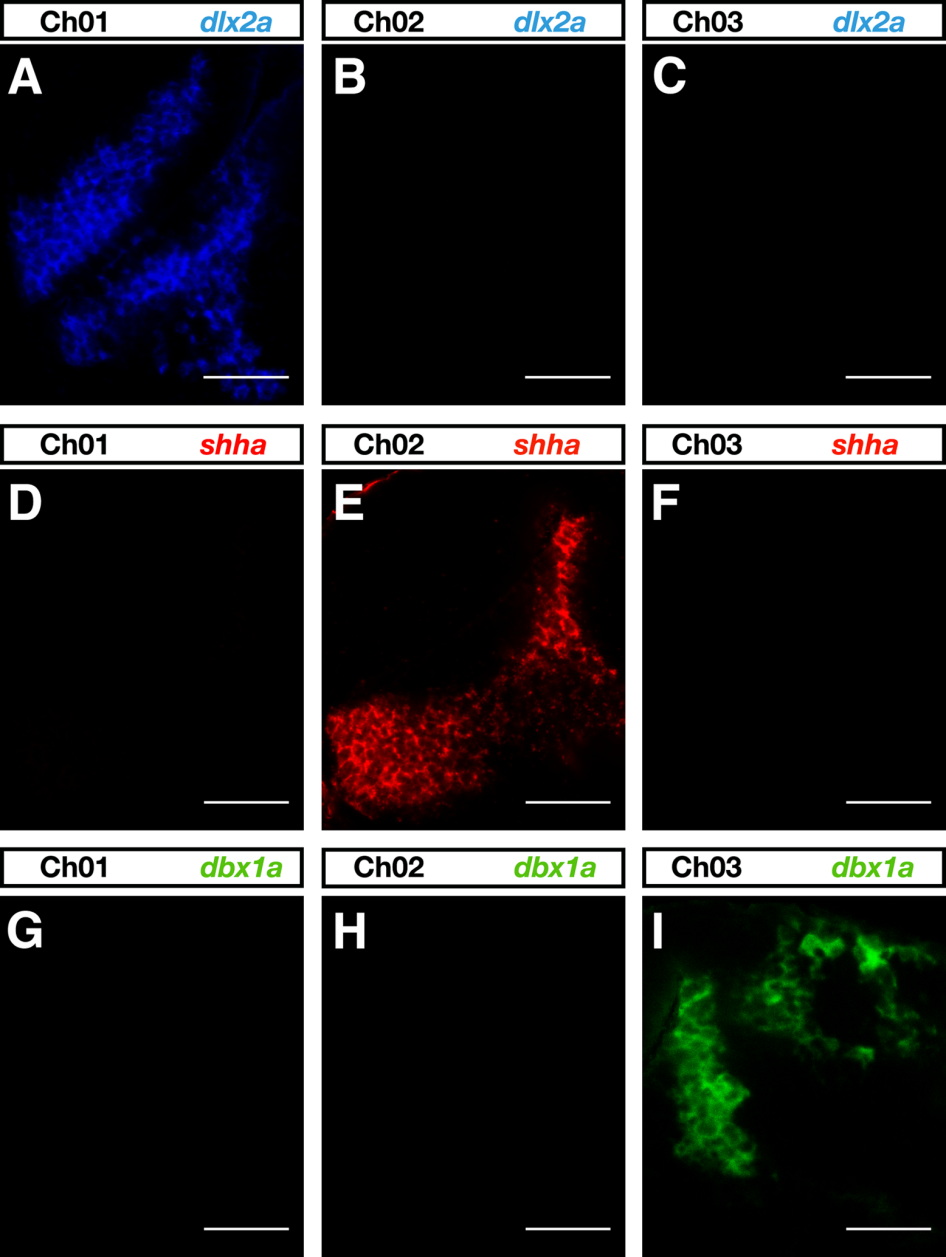
**Test for bleed-through between channels using bench-made tyramides**. **(A-I) **Single-color FISH experiments were performed with digoxigenin-labeled *dlx2a *(A-C), dinitrophenyl-labeled *shha *(D-F) and fluorescein-labeled *dbx1a *(G-I) RNA probes and visualized by FAM-, TAMRA- and DyLight633- tyramide, respectively. Lateral views of 1-dpf forebrains are shown with anterior to the left. Only the appropriate detection channel showed a bright signal and no significant bleed-through between channels was observed. Images were recorded with identical settings to those of Figure 7 on a LSM510 microscope (Carl Zeiss) and false colored in ImageJ. Scale bar = 50 μm.

For colocalization studies it is desirable to simultaneously visualize the expression patterns of multiple genes in the same tissue or embryo preparation. The chosen bench-made fluorescent tyramides match the common 488 nm, 543 nm, and 633 nm excitation laser lines, providing substrate combinations suitable for multicolor experiments. A prerequisite for the use of TSA substrates in multicolor FISH is that there is no substantial bleed-through between the different detection channels. We performed single color FISH experiments with specific probes for *dlx2a *(Figure 4A-C), *shha *(Figure 4D-F), and *dbx1a *(Figure 4G-I) and detected each transcript with a different bench-made tyramide substrate. The expression pattern of each gene was recorded in all three channels. A bright gene-specific signal was visualized only in the detection channel appropriate for the chosen tyramide substrate while significant bleed-through in the other two channels was not detected (Figure 4).

### Effective inactivation of antibody-POD conjugate

A crucial step in multicolor FISH is the inactivation of the antibody-POD conjugate after each round of antibody detection and fluorescent tyramide deposition. Incompletely inactivated POD will generate a fluorescence signal in the second detection round, leading to re-detection of the first signal in the second fluorescence channel. This can result in detection of false positive overlap.

**Figure 5:**
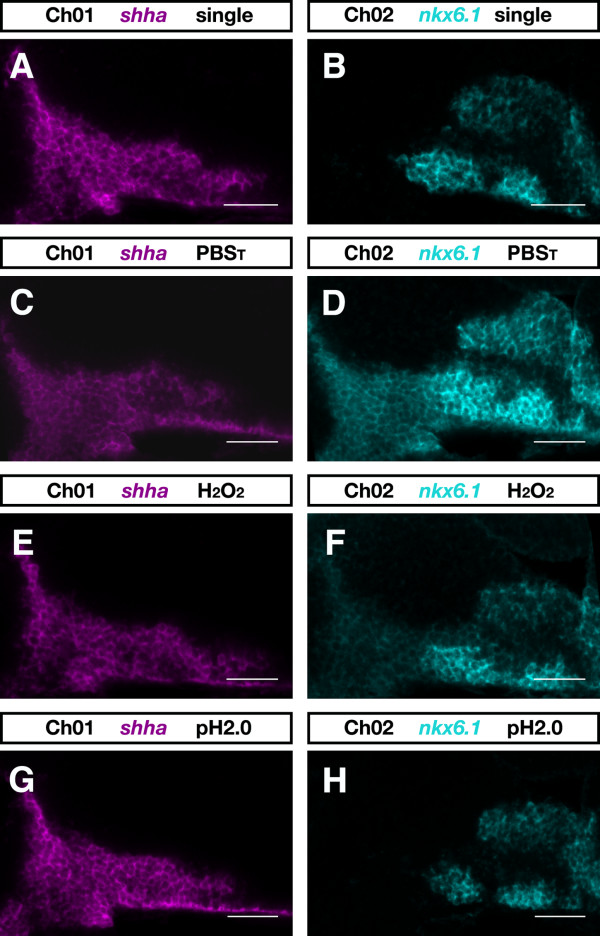
**Inactivation of antibody-POD conjugate**. Zebrafish embryos at 28 hpf were hybridized with dinitrophenyl-labeled *shha *and digoxigenin-labeled *nkx6.1 *RNA probes. **(A, B) **The expression patterns of *shha *(A) and *nkx6.1 *(B) as seen in single-color FISH experiments. In two-color experiments *shha *transcript was detected first using DyLight633-tyramide and *nkx6.1 *transcript was detected subsequently by FAM-tyramide. **(C-H) **Prior to the second round of detection, embryos were incubated for 10 minutes in PBS_T _(PBS plus 0.1% Tween-20) (C, D), PBS_T _containing 6% H_2_O_2 _(E, F), or 100 mM glycine-HCl pH 2.0 (G, H). Single confocal sections of zebrafish brains are shown in the DyLight633-detection channel (Ch01) and in the FAM-detection channel (Ch02) from a lateral view and with anterior to the left. Images were recorded on a LSM510 microscope (Carl Zeiss) and false colored in ImageJ. Scale bar = 50 μm.

To avoid false positive transcript colocalization in multicolor experiments, we compared the efficiency of a method widely used for POD inactivation and one proven to be effective for inactivation of alkaline phosphatase [27,28]. Zebrafish embryos at 28 hpf were simultaneously hybridized with a dinitrophenyl-labeled *shha *and a digoxigenin-labeled *nkx6.1 *antisense RNA probe (Figure 5; Additional file 1). The *shha *transcript was detected first using DyLight633-tyramide and the *nkx6.1 *transcript was detected subsequently using FAM-tyramide. Prior to the second round of detection, embryos were treated for 10 minutes with either buffer solution (Figure 5C, D) or 6% hydrogene peroxide (H_2_O_2_; Figure 5E, F) or 100 mM glycine-HCl pH 2.0 (Figure 5G, H). Any residual staining of *shha *in the FAM-detection channel would indicate incomplete POD inactivation. As expected, *shha *staining was very prominent in the FAM-detection channel in buffer-treated embryos where no inactivation occurred (Figure 5D). Treatment with 6% H_2_O_2 _only partially inactivated POD activity, since *shha *was still detected in the FAM-detection channel (Figure 5F). In control experiments (Figure 5A, B), we could rule out bleed-through by overlap of the DyLight633 and FAM emission spectra as well as antibody cross-reaction with the other hapten (data not shown). However, no residual *shha *signal was detectable after glycine-HCl pH2 treatment, indicating complete removal of POD activity (Figure 5H). Our results are in accordance with those of Liu and co-workers [29], who quantitatively evaluated the efficacy of a number of POD inhibitors. Therefore, overlapping fluorescence signals as signs of colocalization must be evaluated with special caution when H_2_O_2 _is chosen as POD inhibitor. The strong enhancement of sensitivity, when applying our optimized POD reaction conditions, especially requires an efficient inactivation method.

### Multicolor FISH

**Figure 6:**
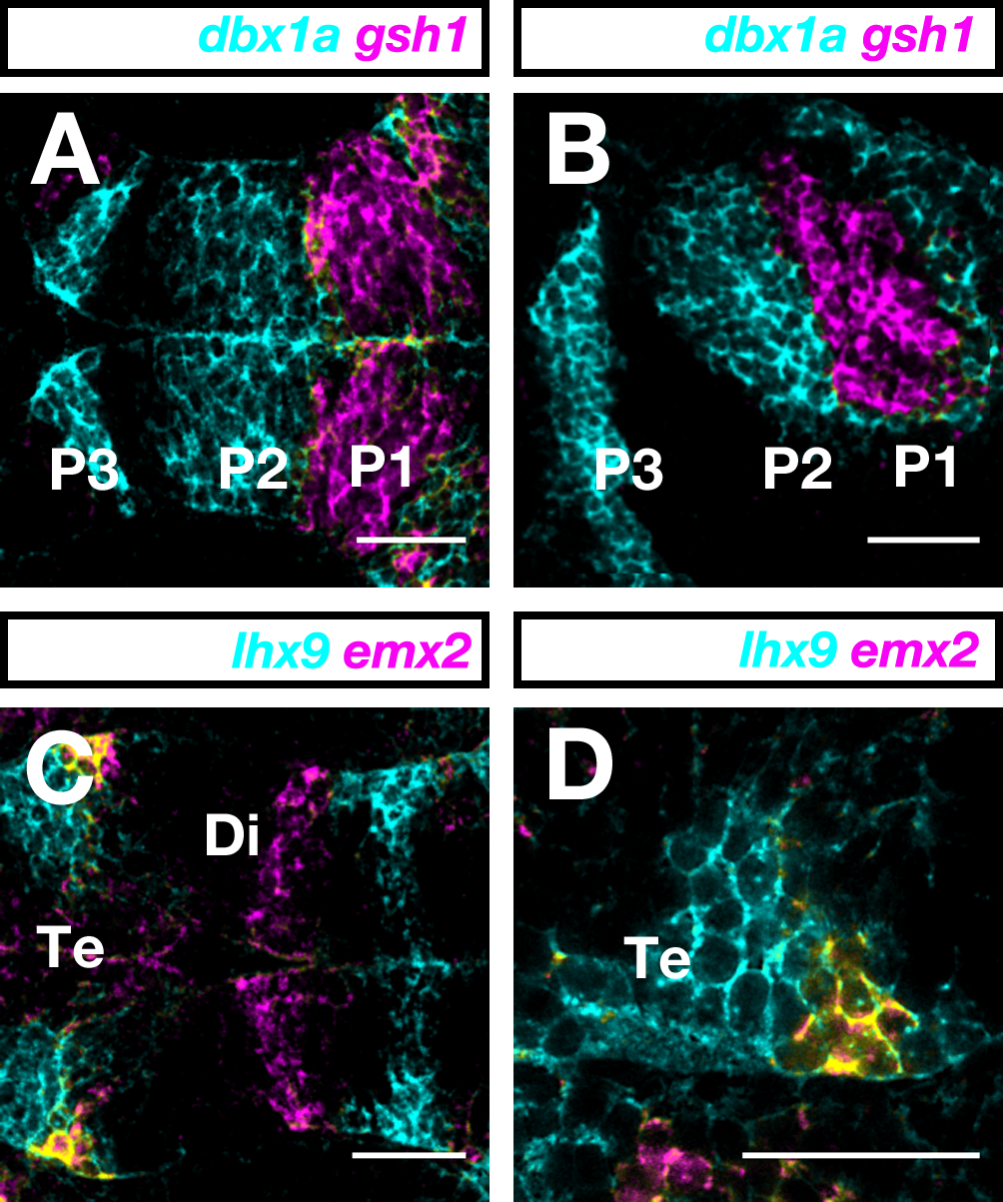
**Two-color FISH**. **(A-D) **Dorsal (A, C, D) and lateral (B) views of 1-dpf zebrafish forebrains with anterior to the left. (A, B) Digoxigenin-labeled *dbx1a *and dinitrophenyl-labeled *gsh1 *RNA probes are shown in cyan and magenta, respectively. (C, D) Digoxigenin-labeled *emx2 *and dinitrophenyl-labeled *lhx9 *RNA probes were visualized in magenta and cyan, respectively. In the telencephalon, *emx2 *and *lhx9 *are co-expressed in a lateral cell cluster (yellow). (D) Higher magnification of the area of colocalization (yellow) in the telencephalon. Photographs were taken on a LSM510 confocal microscope (Carl Zeiss). Images were false-colored with RGB look-up-tables and processed using ImageJ software. Scale bar = 50 μm. Di, diencephalon; P1, P2, and P3, prosomeres 1, 2, and 3; Te, telencephalon.

The introduced optimization steps were prerequisites for successfully performing multicolor FISH and allowed us to resolve forebrain gene expression domains in two- and even three-color FISH experiments. We compared the expression of the homeobox genes *dbx1a *[30] and *gsh1 *[31] in the embryonic diencephalon. Two-color FISH demonstrated that the two genes were expressed in directly adjacent but clearly separate diencephalic domains (Figure 6A, B). According to the prosomeric model, the diencephalon consists of three prosomeres, the pretectum, thalamus, and prethalamus or prosomeres p1, p2, and p3, respectively [2]. While *gsh1 *expression was confined to most of the pretectum (p1), *dbx1a *was prominently expressed in caudal prethalamus (p3) and caudal thalamus (p2) and extending into the rostral pretectum (p1).

To demonstrate the potential of our multicolor FISH procedure to resolve co-localization at the cellular level, we compared expression of *lhx9 *[32] with that of *emx2 *[33] in the telencephalon and diencephalon. In the diencephalon, expression domains of *lhx9 *and *emx2 *were distinct from each other while in the telencephalon a small region of overlap was eminent (Figure 6C). At higher magnification, optical confocal sectioning revealed *lhx9*/*emx2 *colocalization in a small clutch of pallial cells (yellow; Figure 6D). In the diencephalon, the transverse *emx2 *and *lhx9 *domains corresponded to the rostral and caudal thalamus, respectively, suggesting an intraprosomeric molecular subdivision of p2.

**Figure 7:**
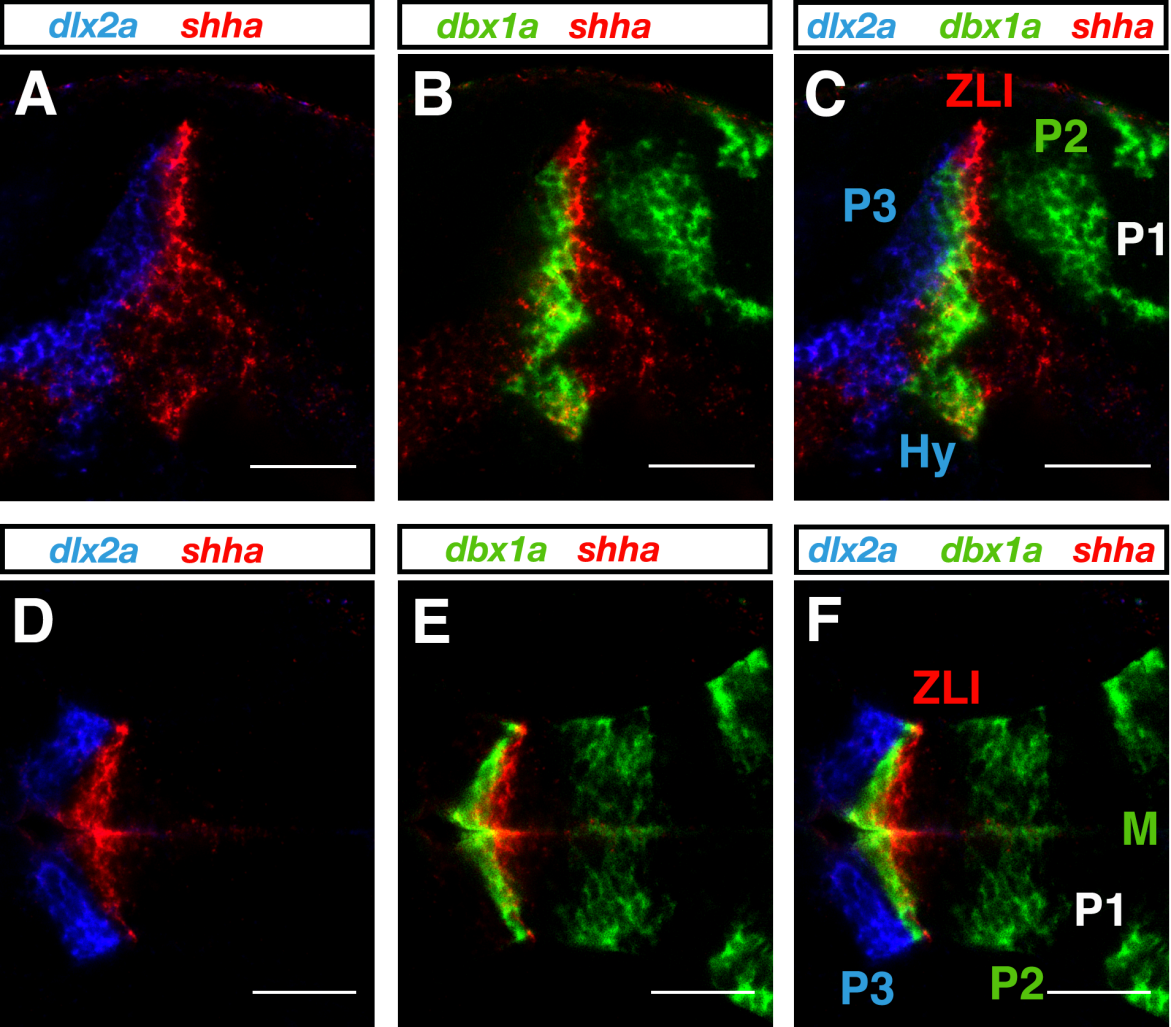
**Three-color FISH**. Embryos at 1 dpf were hybridized simultaneously with fluorescein-labeled *dbx1a*, dinitrophenyl-labeled *shha *and digoxigenin-labeled *dlx2a *RNA probes, sequentially detected and visualized using DyLight633-, TAMRA- and FAM-tyramide, respectively. **(A-F) **In lateral (A-C) and dorsal (D-F) views with anterior to the left, *dbx1a*, *shha *and *dlx2a *expression is shown in green, red and blue, respectively. Overlays of two (A, B, D, E) or all three (C, F) different channels of the same confocal plane are shown. Photographs were taken on a LSM510 confocal microscope (Carl Zeiss). Images were false-colored with RGB look-up tables and processed using ImageJ software. Scale bar = 50 μm. Hy, hypothalamus; M, midbrain; P1, P2, and P3, prosomeres 1, 2, and 3; ZLI, zona limitans intrathalamica.

Similarly to *dbx1a *(Figure 6A, B), *shha *[34] and *dlx2a *[35] are expressed in distinct transverse and longitudinal forebrain expression domains [10,12]. We compared their expression in a triple-color FISH experiment (Figure 7). Forebrain expression of *dlx2a *was confined to the telencephalon, hypothalamus and prethalamus, *dbx1a *to diencephalic prosomeres and midbrain, and *shha *to zona limitans intrathalamica and basal forebrain (shown in Figures 4 and 7 in blue, green and red, respectively). In a dorsal view, overlay of the different channels showed that forebrain expression domains of the three genes did not show any overlap except for the p2/p3 border region, the zona limitans intrathalamica (Figure 7F). Prethalamic *dlx2a *expression stopped short at the p2/p3 border and abutted *shha *expression (Figure 7A, D). At the zona limitans intrathalamica, *shha *and *dbx1a *slightly overlapped and co-expression appeared in yellow (Figure 7E). In a lateral view, overlap of *shha *and *dbx1a *could be observed in the basal plate of p3 corresponding to the posterior tuberculum ventralis (Figure 7B, C). The expression patterns visualized here by multicolor FISH (Figures 6 and 7) are consistent with a three-prosomere organization of the embryonic zebrafish diencephalon, supporting a conservation of diencephalic subdivisions between anamniotes and amniotes.

Multicolor FISH relies on the hybridization with a mixture of differently haptenylated RNA probes. In our hands, digoxigenin- and dinitrophenyl-labeled RNA probes provided comparable sensitivity, while fluorescein-labeled probes were significantly less sensitive. We thus routinely combined digoxigenin- and dinitrophenyl-labeled probes in two-color FISH and used the fluorescein label for the strongest expressed transcript in three-color experiments. In two-color FISH, dinitrophenyl was preferably detected first using DyLight633-tyramide followed by digoxigenin with FAM-tyramide. In case three different transcripts were visualized, the fluorescein probe was detected first using DyLight633-tyramide followed by dinitrophenyl with TAMRA-tyramide and digoxigenin with FAM-tyramide.

We chose not to use TSA-POD reaction times longer than 30 minutes for any probe because prolonged staining could lead to significant amplification of background noise. To achieve a high quality staining within a relatively short reaction time, optimized reaction conditions and adjusted probe concentrations should be determined. As a rule of thumb, we routinely increased RNA probe concentration by about 30% for TSA-POD detection compared to chromogenic alkaline phosphatase/NBT/BCIP staining. Higher increases in probe concentration included the risk of background amplification and negative effects on signal-to-noise ratio. In our hands, expression patterns that could be visualized within 5 to 6 hours using standard chromogenic staining were relatively easy to be visualized by the TSA system. For example, *dlx2a*, *dbx1a *and *shha *probes could be routinely visualized in high quality by chromogenic staining within 3 to 4 hours and by the TSA-POD system within 20 minutes. Gene expression patterns that took 8 to 10 hours staining time for proper chromogenic detection were more problematic but could still be visualized by FISH (for example, the *tyrosine hydroxylase *gene).

## Conclusions

In total we developed a sensitive procedure for multicolor fluorescent detection of up to three different mRNA species in whole zebrafish embryos at cellular resolution. We used this method for visualization of abutting and overlapping transcript distribution of regulatory genes in the developing forebrain. In a separate study we successfully used our newly developed technique for correlation of expression patterns of a great number of regulatory genes to highlight potential molecular subdivisions in the embryonic zebrafish diencephalon in the absence of prominent morphological features at early developmental time points (unpublished data). Likewise, our method can be applied to any other tissue in the zebrafish embryo for detection of different transcript combinations. The key to successfully perform multicolor FISH was the substantial improvement of hybridization, POD reaction and inactivation conditions by the application of viscosity-increasing polymers, POD accelerators and glycine-HCl treatment. These optimization steps have the potential to improve sensitivity of FISH procedures in other model organisms as well. Similarly, our optimized POD-TSA conditions may be beneficial for any other application where TSA is applied in POD-mediated detection, including immunocytochemistry and immunohistochemistry. Thus, it is to be expected that our improvements will be carried over to other model organisms and TSA applications for increasing sensitivity and signal-to-noise ratio.

## Materials and methods

### Zebrafish maintenance

Wild-type zebrafish embryos were obtained by natural mating and raised under standard conditions at 28.5°C until fixation at the desired stage. Embryos were staged in hours and days post-fertilization (hpf and dpf) according to [36]. All experiments were done on fixed specimens and in accordance with ethical permits obtained from the Stockholms södra djurförsöksetiska nämnd and jordbruksverket.

### RNA probes

RNA probes were generated by *in vitro *transcription following a protocol from [6,7] and using either digoxigenin-11-UTP (Roche Scandinavia: Bromma, Sweden 11209256910) or fluorescein-12-UTP (Roche 11427857910) or dinitrophenyl-11-UTP (Perkin Elmer: Waltham, MA, USA NEL555001EA) as a label. RNA probes were column purified using a RNA Probe Purification Kit (Omega Bio-Tek: Norcross, GA, USA R6249-02) following the manufacturer's instructions. The following cDNA templates for generating RNA probes were used: *dbx1a *[30], *nkx6.1 *[37], *shha *[34], *dlx2a *[35], *lhx9 *[32], *emx2 *[33], and *gsh1*[31].

### Embryo pretreatment

All steps were performed at room temperature unless stated otherwise. For permeabilization, fixed zebrafish embryos were kept in 100% methanol for at least 30 minutes at -20°C. To further increase accessibility, embryos were treated with 2% H_2_O_2 _in methanol for 20 minutes. Subsequently, embryos were rehydrated and digested with proteinase K as described by [7]. Embryos were stored in hybridization buffer (Hb4: 50% deionized formamide, 5×SSC, 5 mg/ml torula RNA (Sigma R-6625), 50 mg/ml heparin sodium salt, 0.1% Tween-20) at -20°C until usage.

### Hybridization

Embryos were prehybridized in Hb4 for 1 h at 60°C. RNA probes were diluted in hybridization buffer (Hb4D5) containing 5% (v/v) dextran sulfate (stock solution 50% w/v; Sigma: St. Louis, MO, USA, D6001) and denatured for 5 minutes at 80°C. The prehybridization buffer was exchanged with the probe mix and hybridization was performed at 60°C overnight (minimum of 15 h). Unbound, excessive probe was removed by two washes in 50% formamide in 2×SSC 0.1% Tween-20 for 30 minutes, one wash in 2×SSC 0.1% Tween-20 for 15 minutes, followed by two washes in 0.2×SSC 0.1% Tween-20 for 30 minutes. All washing solutions were pre-warmed to 60°C. Subsequently, embryos were rinsed twice with PBS_T _(PBS plus 0.1% Tween-20) at room temperature.

### Antibody incubations

RNA probes were detected by antibodies coupled to POD and directed against the adequate hapten in consecutive rounds of detection. Embryos were blocked in 8% normal sheep serum (Sigma S2263) in PBS_T _for 1 h at room temperature with gentle agitation and then incubated with the appropriate antibody overnight without agitation at 4°C. All antibodies were diluted in blocking solution. Sheep-anti-digoxigenin-POD Fab fragments (Roche 11207733910) were diluted 1:500, anti-dinitrophenyl-POD (PerkinElmer TSA Plus DNP System NEL747A001KT) was diluted 1:100 and the rabbit-anti-fluorescein/Oregon Green 488-POD (Molecular Probes: Eugene, OR, USA A21253) was diluted 1:500. To remove unbound antibody, embryos were washed six times in PBS_T _for 20 minutes at room temperature with gentle agitation.

### TSA reaction

Prior to the TSA reaction, embryos were rinsed twice in 100 mM borate pH 8.5 plus 0.1% Tween-20. The TSA reaction buffer was prepared freshly each time and contained 100 mM borate pH 8.5, 2% dextran sulfate, 0.1% Tween-20 and 0.003% H_2_O_2_. As an accelerator for the POD reaction, 4-iodophenol (Fluka: Buchs, Switzerland 58020) or vanillin (Sigma V110-4) were added to the reaction buffer at a concentration of 150 to 450 μg/ml. Commercial tyramide reagents used were: fluorescein tyramide reagent (PerkinElmer SAT701), cyanine-3 tyramide reagent (PerkinElmer SAT704A), and cyanine-5 tyramide reagent (PerkinElmer SAT705A). Synthesized tyramide reagents used were carboxyfluorescein tyramide (FAM-tyramide), carboxytetramethylrhodamine tyramide (TAMRA-tyramide) and DyLight633 tyramide. All commercial tyramide reagents were diluted 1:100 and synthesized tyramide reagents were diluted 1:250 in TSA reaction buffer (except for the DyLight633-tyramide, which was diluted 1:167).

The TSA reaction was allowed to proceed for 15 to 30 minutes protected from light and without agitation. Thereafter, samples were rinsed thoroughly four times in PBS_T _with inverting tubes several times at each washing step. When more than one probe was detected, the POD activity of the previous antibody was inactivated by treatment of embryos with 100 mM glycine pH 2.0 plus 0.1% Tween-20 for 10 minutes [6]. Subsequently, embryos were washed four times for 5 minutes in PBS_T _with gentle agitation followed by the next round of detection.

### Tyramide synthesis

To synthesize tyramide reagents [26], the following succinimidyl esters were used for conjugation with tyramine (Sigma-Aldrich: St. Louis, MO, USA T2879): 5-(and-6)-carboxyfluorescein succinimidyl ester (FAM-SE; Molecular Probes C-1311), 5-(and-6)-carboxytetramethylrhodamine succinimidyl ester (TAMRA-SE; Molecular Probes C-1171) and DyLight 633 N-hydroxysuccinimide ester (DyLight633-SE; Pierce: Rockford, IL, USA 46414).

Each succinimidyl ester was dissolved at a concentration of 10 mg/ml in dimethylformamide (Sigma-Aldrich D4551). A 10× stock solution of tyramine hydrochloride was prepared at a concentration of 100 mg/ml in dimethylformamide. The tyramine working solution was prepared from the 10× stock with dimethylformamide and 10 μl triethylamine (>99%; Sigma-Aldrich T0886) per milliliter of tyramine working solution was added. For synthesis of tyramide conjugates, the tyramine working solution and the respective succinimidyl esters were mixed at a 1:1.1 equimolar ratio. The reaction was allowed to proceed in the dark without agitation for 2 hours at room temperature. The tyramide products were diluted with absolute ethanol to a final concentration of 1 mg/ml and were stable for at least 3 years at -20°C.

### Imaging

For images of single-color fluorescent embryos taken either with a Carl Zeiss Axiocam color-camera or a Hamamatsu ORCA-ER black-and-white camera under a Zeiss Axioplan2 microscope, embryos were mounted in 75% glycerol in PBS_T _pH 8. Black and white pictures were false-colored using ImageJ software; no further adjustments or image processing was done. For pictures of multicolor-fluorescent embryos recorded with a Zeiss LSM510 confocal microscope (Carl Zeiss) embryos were mounted in 75% glycerol in TNT_W _pH 8 (100 mM Tris pH 8, 150 mM NaCl, 0.1% Tween-20) containing 1% low melting agarose. Opensource ImageJ software was used for image processing to adjust outliers and intensity levels before running a Gaussian smoothening filter. Figure assembly was done with Adobe Photoshop software. The signal-to-noise ratios were determined with help of the ROI manager of ImageJ. An area with clear signal was selected and its mean intensity value was compared to the mean intensity of an identical area lying close to the expression site within the embryo. This procedure was repeated for three different expression sites to determine the mean value of three data points. For each gene the same expression sites were selected in different embryos.

## Abbreviations

dpf: days post-fertilization; FAM: carboxyfluorescein; FISH: fluorescent *in situ *hybridization; hpf: hours post-fertilization; PBS_T_: phosphate buffered saline plus 0.1% Tween-20; POD: peroxidase; TAMRA: carboxytetramethylrhodamine; TSA: tyramide signal amplification.

## Competing interests

The authors declare that they have no competing interests.

## Authors' contributions

GL and IS did the experiments. GL and GH designed the project and all three authors helped to draft and write the manuscript. All authors read and approved the final version of the manuscript.

## Supplementary Material

Additional file 1 **Three-dimensional reconstruction of *shha *and *nkx6.1 *brain expression**. Three-dimensional reconstruction of *shha *(cyan) and *nkx6.1 *(magenta) expression in the 1-dpf zebrafish brain generated with the ImageJ three-dimensional viewer plugin. Both *shha *and *nkx6.1 *are expressed in the basal posterior diencephalon and midbrain tegmentum. In other rostral brain regions only *shha *or *nkx6.1 *are expressed. Click here for file
